# Intracellular Reactive Oxygen Species Mediate the Therapeutic Effect of Induced Pluripotent Stem Cells for Acute Kidney Injury

**DOI:** 10.1155/2020/1609638

**Published:** 2020-03-26

**Authors:** Shun Wang, Xiaoyu Tian, Yijun Li, Rong Xue, Haochen Guan, Meng Lu, Huijun Xu, Zhibin Ye, Sifeng Chen, Meng Xiang

**Affiliations:** ^1^Department of Physiology & Pathophysiology, School of Basic Medical Sciences, Fudan University, Shanghai, China; ^2^Department of Nephrology, Huadong Hospital Affiliated with Fudan University, Shanghai, China

## Abstract

**Aims:**

Treatment for acute kidney injury (AKI) is challenging. Induced pluripotent stem cells (iPSCs) have great therapeutic potential. This study sought to determine whether iPSCs attenuate AKI and the role of reactive oxygen species (ROS).

**Results:**

We intravenously injected isogenic iPSCs into mice 2 h after renal ischemia-reperfusion injury (IRI). The cells were selectively trafficked to ischemia/reperfusion-injured kidney where they decreased kidney ROS and inflammatory cytokines and improved kidney function and morphology. Pretreating the cells with ROS inhibitors before administration decreased iPSC engraftment and abolished the protective effect of iPSCs. In contrast, pretreating iPSCs with hydrogen peroxide increased iPSC engraftment and therapeutic effect. Although the intravenously administered iPSCs trafficked to the IRI kidney, the cells did not differentiate into proximal or distal tubular epithelial cells. *In vitro*, the capabilities of the iPSC-released substances to promote proliferation and decrease apoptosis of renal epithelial cells were increased by ROS pretreatment of iPSCs. Moreover, pretreatment of the iPSCs with ROS inhibitor had the opposite effect. Similarly, moderate concentrations of ROS increased while ROS inhibitors decreased iPSC mobility, adhesion to the extracellular matrix, and mitochondrial metabolism. *Innovation and Conclusion*. iPSCs decreased renal ischemia/reperfusion injury mainly through iPSC-released substances. The therapeutic effect, mitochondrial metabolism, mobility, and kidney trafficking of iPSCs were ROS dependent.

## 1. Introduction

Renal ischemia-reperfusion injury (IRI) is a common clinical problem associated with a high incidence of morbidity and mortality [1, 2]. It is often a secondary result of certain diseases and procedures such as trauma, hypovolemic shock, severe cardiovascular disease, renal transplantation, partial nephrectomy, cardiopulmonary bypass, and sepsis. Treatment options for renal IRI remain limited to supportive measures and renal replacement therapy. Consequently, the development of new therapies is critical.

Induced pluripotent stem cells (iPSCs) are stem cells reprogrammed from somatic cells with unlimited proliferative potential; they hold great therapeutic potential for most major diseases, including acute kidney injury (AKI) [3, 4]. iPSCs are easily accessible and abundant sources of regenerative cells available for real-time and autologous use. Autologous iPSCs can be induced, abundantly cultured, and stocked for future applications [5]. Furthermore, they can achieve therapeutic effect through multiple mechanisms, including endo/paracrine [6] and differentiating into the cells of all three germ layers [7] in response to the needs of tissue microenvironment.

The possible formation of teratomas is a hurdle when using iPSCs *in vivo*. Using 53 conditions and animal disease models, we demonstrated our hypothesis that congregated iPSCs control the microenvironment *in vivo* and generate teratomas while disseminated iPSCs are controlled by the microenvironment *in vivo* and act according to local requirements. Disseminating iPSCs intravenously or topically are crucially important for cell therapies not only because of their convenience but also because teratomas are thereby easily and completely avoided [8]. Moreover, other safety shields such as embedded inducible suicide genes in the iPSCs are also available. Intravenously administered iPSCs constitute ideal autologous candidates for cell therapies in AKI.

The microenvironment is critical for stem cell differentiation and the maintenance of pluripotency for which a specific culture medium is required. By delicately controlling the culture conditions, iPSCs can be directed to differentiate into lineage-specific cells [9–11]. Tissue microenvironments change over time in response to the nature of the etiological factors of disease and recovery. Reactive oxygen species (ROS) are the side products of many molecular processes including normal mitochondrial respiration [12]. Mitochondria may have a crucial role in reprogramming iPSCs, in the maintenance of a pluripotent state, and in differentiation [13, 14]. iPSCs maintain a low level of ROS production [15]. However, intracellular ROS production increases dramatically when the cells are in the process of monolayer differentiation or embryoid body differentiation [15]. Persistent treatment with hydrogen peroxide (H_2_O_2_, 100 *μ*mol/L) injured iPSCs *in vitro*. However, whether ROS inside iPSCs play a role in the therapeutic potential of iPSCs *in vivo* is unknown.

ROS production in organs increases significantly during many pathophysiological processes such as IRI and inflammatory respiratory burst, causing direct and indirect cellular damage [16]. Antioxidant therapy is protective against IRI-mediated oxidative damage in different experimental models [17, 18]. Administered iPSCs will inevitably come in contact with ROS. Thus, it is important to know whether extracellular ROS will affect the mitochondrial respiration and energy production of iPSCs and the ability of the cells to treat renal IRI.

This study sought to determine whether iPSCs attenuate AKI and the possible mechanisms involved, especially the role of reactive oxygen species (ROS).

## 2. Materials and Methods

### 2.1. Mouse Strain and Reagents

Male C57BL/6 mice were purchased from the Shanghai SLAC Laboratory Animal Co., Ltd. (Shanghai, China) and housed in an animal facility at Fudan University. Animal protocols were approved by the University of Fudan animal care committee adapted from the criteria of the National Research Council's Guide for the Care and Use of Laboratory Animals. Mice were 8 to 10 weeks of age at the time of the experiments. They were fed standard rodent chow and given water *ad libitum*.

Detailed information on primer sequences and key resources is provided in Supplemental [Supplementary-material supplementary-material-1].

### 2.2. iPSC Culture and Purification

iPSCs were induced in house to ensure that the cells were syngeneic to the C57BL/6 recipients and identified as iPSCs using classic criteria based on morphology, biological marker, and teratoma formation assays as described in the literature [13]. The iPSCs were maintained on mitomycin C-treated mouse embryonic fibroblast (MEF) feeder cells in standard ESC medium containing 10% KSR. Prior to injection, the iPSCs and feeder cells were dissociated into single cells with 0.25% trypsin-EDTA after which they were washed three times with PBS. The cells were suspended with an iPSC culture medium and plated onto a culture dish pretreated with 0.2% gelatin. After incubation at 37°C for 30 min, the detached cells (mainly feeder cells) were removed by aspirating the supernatant. iPSCs were more likely to adhere, thereby remaining on the dish. iPSCs weakly attached to the dish were then detached and blown with PBS. After centrifugation, the cells were resuspended in PBS for injection. Any contaminated MEFs were unable to survive due to the mitomycin-c pretreatment.

### 2.3. Kidney Ischemia-Reperfusion Injury

Kidney IRI was induced according to the literature [19]. In brief, male C57BL/6 mice were anesthetized with an intraperitoneal injection of sodium pentobarbital. Kidneys were exposed by bilateral flank incisions and subjected to right-sided nephrectomy, followed by left-sided renal ischemia induced by clamping the renal artery with a nontraumatic microvessel clamp (size B-1 V; S & T AG, Neuhausen, Switzerland) for 55 min. After the clamp was released and the reperfusion was confirmed visually and Laser Doppler blood flow measurement (Moor Instruments, Devon, UK) (Supplemental Fig. [Supplementary-material supplementary-material-1]), the bilateral flank incisions were closed with a 3-0 silk suture. All mice received 30 *μ*L saline/g of body weight subcutaneously after the surgery to replenish fluid loss. One hour after the procedure, iPSCs (3 × 10^6^/kg body weight, *n* = 20), H_2_O_2_-treated iPSCs (3 × 10^6^/kg body weight, pretreated with 100 *μ*M H_2_O_2_ for 2 h, *n* = 20), NAC-treated iPSCs (3 × 10^6^/kg body weight, pretreated with 100 *μ*M NAC for 2 h, *n* = 20), or an equal volume (5 mL/kg body weight, *n* = 20) of phosphate buffered saline (PBS, 100 *μ*L) was administered via the tail vein. Sham operations (*n* = 20) were conducted using the same procedure without clamping the left renal artery. At 24 h after reperfusion, half of the mice were sacrificed with an overdose of pentobarbital. At 48 h, the remaining mice were sacrificed. Blood samples for blood creatinine and blood urea nitrogen (BUN) measurements were collected directly from the heart. The left kidney was excised and divided evenly into four quarter pieces. Three pieces were used for the measurements of ROS, cytokines, and iPSC quantification in the kidney. The remaining piece was fixed in 4% buffered paraformaldehyde and 1% methanol for histological examination. Additional kidney IRI animal models of the above five different groups were performed to determine the glomerular filtration rate (GFR; *n* = 3 per group) and to determine if iPSCs differentiate into renal epithelial cells in vivo (*n* = 5 per group).

### 2.4. Histological Examination

The kidney tissue was embedded in paraffin. Four-micrometer sections were subjected to hematoxylin and eosin (H&E) staining. Histologic analyses were performed on images taken with a DMR Leica microscope (Leica Microsystems, Wetzlar, Germany). The mean of at least 10 fields (200x) of each kidney was scored for tubular cell necrosis, cytoplasmic vacuole formation, hemorrhaging, and tubular dilation. A scale ranging from 0–4 (0 = normal, 1 = minimal damage (0%–5% injury), 2 = mild damage (5%–25% injury), 3 = moderate damage (25%–75% injury), and 4 = severe damage (75%–100% injury)) was used. Analyses were performed on blinded sections by two independent observers.

### 2.5. Measurement of Blood Creatinine and BUN

The blood creatinine and BUN levels were evaluated using a creatinine assay kit and BUN assay kit, respectively, according to the manufacturer's instructions (Jiancheng Bioengineering Institute, Nanjing, China).

### 2.6. iPSC Quantification in the Kidney

Plasmid 20321 (plasmid TetO-FUW-OSKM from Addgene, Watertown, MA, USA) was used to produce lentiviruses for the induction of pluripotent stem cells from mouse fibroblast cells. Given that the *Tet* gene is an external gene, it can be used as a target for iPSC identification and quantification.

PCR was performed using chromosomal DNA isolated from kidney tissue as templates. *GADPH* (glyceraldehyde-3-phosphate dehydrogenase) expression was used as an internal control.

### 2.7. Measurement of Cytokine Expression in Injured Kidney

Messenger RNA of interleukin-1*β*, C-X-C motif chemokine ligand 1 (CXCL1), interleukin-6, monocyte chemotactic protein 1 (MCP-1), tumor necrosis factor-*α* (TNF-*α*), and interleukin-10 were measured by quantitative reverse transcription PCR (RT-qPCR) at 24 h and 48 h after renal IRI. In brief, total RNA from kidney tissue was isolated using TRIzol® reagent following the manufacturer's instructions (Invitrogen/Thermo Fisher Scientific, Carlsbad, CA). RNA concentration and purity were determined using a NanoPhotometer® (Implen GmbH, Munich, Germany) at a wavelength of 260/280 nm. Total RNA was then reverse-transcribed using a SuperScript® Preamplification Kit (Life Technologies/Thermo Fisher Scientific, Carlsbad, CA, USA). Primers (Supplemental [Supplementary-material supplementary-material-1]) for qRT-PCR SYBR® Green assays were designed using Primer 5.0 software. The amplification reactions proceeded as follows: 40 cycles of 20 s at 95°C, 45 s at 57°C, 30 s at 72°C, after initial denaturation at 95°C for 5 min with 1X SYBR® Green PCR Master Mix (Applied Biosystems/Thermo Fisher Scientific, Foster City, CA, USA). Primers and reaction conditions were vetted by melting curve analysis.

### 2.8. Measurement of ROS Production in Kidney Tissue

ROS production was measured using a fluorescence assay as described previously [20]. Briefly, 50 mg of kidney tissue was homogenized in 1 mL PBS. Then 100 *μ*L of sample and 10 *μ*L of DHE (final concentration 5 *μ*mol/L) were added to a well of a 96-well plate and incubated for 30 min at 37°C and analyzed (excitation 300 nm/emission 610 nm) on a fluorescence plate reader (Synergy H1, BioTek Instruments Inc., Winooski, VT, USA). ROS concentration was calculated based on comparison to a predetermined H_2_O_2_ standard curve.

### 2.9. iPSC Trafficking and Differentiation in the Kidney

To track iPSCs *in vivo*, cells were labeled with the red fluorescent dye PKH26 (Sigma-Aldrich, St. Louis, MO) prior to a bolus intravenous injection via tail vein. The kidney tissue was harvested and fixed in PBS containing 4% formaldehyde and 1% methanol for 24 h. They were dehydrated in the order of 10%, 20%, and 30% sucrose and then embedded in optimum cutting temperature compound before being frozen in liquid nitrogen. Frozen sections (8 *μ*m thick) of the kidney block were cut, mounted on slides, counterstained with DAPI (4′,6-diamidino-2-phenylindole dihydrochloride) and immediately photographed for blue and red fluorescence under an inverted fluorescent microscope (Leica SCN400, Leica Microsystems, Wetzlar, Germany). The location of the photographing was marked for future observation of the same area because the following procedure would quench the red fluorescent dye PKH26.

The frozen sections were then microwaved for 5 min in retrieval solution and cooled at 22°C for 1 h. To stain proximal and distal renal tubular cells, the cryosections of kidney were blocked with 5% donkey serum (NC9624464, Jackson ImmunoResearch Labs, Waltham, MA) and incubated with monoclonal anti-aquaporin 1 (1 : 100 dilution; Abcam Inc., Cambridge, UK) or anti-calbindin D28K (1 : 100 dilution; Santa Cruz Biotechnology Inc., CA) antibody overnight at 4°C, followed by incubation with donkey anti-mouse IgG (1 : 500, Jackson ImmunoResearch Labs) for 1.5 h at 22°C. DAPI was used again to counterstain the nuclei. The area previously photographed for labeled cells in frozen sections was observed using an inverted fluorescent microscope (Leica SCN400, Leica Microsystems, Wetzlar, Germany).

### 2.10. Transcutaneous Measurement of GFR

The measurement was performed as described previously [21], based on measuring the excretion kinetics of a single intravenous bolus of fluorescein isothiocyanate- (FITC-) sinistrin. A miniaturized device (NIC-Kidney, Medibeacon GmbgH, Mannheim, Germany) was fixed with a tape on the previously shaved back of male C57BL/6 mouse. The device was equipped with two light-emitting diodes that transcutaneously excite FITC-sinistrin at 480 nm and a photodiode to detect the emitted light signal at 521 nm. The FITC-sinistrin (7.5 mg/100 g body weight) was injected via tail vein after the background signal of skin was collected for 5 min. Data were then collected (60 measurements/min) for additional 90 min and stored in the internal memory of the microcontroller before detaching the device. The FITC-sinistrin half-life (*t*1/2) was calculated using MPD Studio software ver.RC17 (Medibeacon GmbgH). The GFR was then determined using the following equation [21].

(1) $$\begin{array}{c} \text{GFR }\left[\mu \text{L}\cdot \min\cdot 100\text{ g bw}\right]=\frac{14616.8\;\left[\mu \text{L}/100\text{ g bw}\right]}{t\;1/2\left(\text{ FITC}‐\text{sinistrin}\right)\left[\min\right]\;}. \end{array}$$

### 2.11. Measurement of Intracellular ROS

iPSCs with or without pretreatment with H_2_O_2_ (100 *μ*M) or NAC (100 *μ*M) for 30 min were incubated with DCFH-DA for 30 min at 37°C, analyzed on a fluorescence plate reader (BioTek Instruments), and quantified based on an H_2_O_2_ standard curve. The experiments were repeated three times.

### 2.12. Cell Viability, Adhesion, and Migration

iPSCs pretreated with 100 *μ*M H_2_O_2_ or 100 *μ*M NAC for 2 h, respectively, were added to a 96-well plate (1 × 10^4^ cells per well). iPSCs without treatment served as controls. Twenty-four hours after incubation, cell viability was measured using Cell Counting Kit-8 (CCK-8; Dojindo Molecular Technologies Inc., Shanghai, China) according to the manufacturer's instructions. The experiment was repeated five times.

A cell adhesion assay was performed according to the procedures described by Iwai et al. with some modifications [22]. In brief, iPSCs were labeled with PKH 26 red fluorescent dye (Sigma-Aldrich/Merck Group, St. Louis, MO, USA) at 37°C and pretreated with 100 *μ*M H_2_O_2_, or 100 *μ*M NAC for 2 h. A suspension of the labeled cells (5000 cells/well) was placed in a Matrigel™-coated 96-well plate (BD Biosciences, Franklin Lakes, NJ, USA). After 4 h incubation, nonadherent cells were removed by gently washing the plate 3 times with *PBS*. Adherent cells were photographed with a fluorescence microscope (Leica) and counted. The assays were repeated 10 times independently.

To measure cell migration, iPSCs were pretreated with 100 *μ*M H_2_O_2_ or 100 *μ*M NAC for 2 h, respectively, after which the cells (1 × 10^4^ per well) were labeled with the red fluorescent dye PKH26 (Sigma-Aldrich) and plated on 24-well Transwell™ Permeable Supports with porous filters (Corning Inc., Corning, NY) and incubated for 16 h. The fluorescence positive cells that migrated through the membrane were photographed with a fluorescence microscope and analyzed with ImageJ software (the National Institutes of Health, Bethesda, MD, USA). The experiment was repeated 10 times independently.

### 2.13. Mitochondrial Bioenergetics Assay

The impact of H_2_O_2_ and NAC treatment on mitochondrial energetics in iPSCs was evaluated using mitochondrial bioenergetics kit according to the manufacturer's instruction (Agilent Technologies Inc., Santa Clare, CA). Briefly, cells (30,000 iPSCs/well) were seeded in 96-well Seahorse tissue culture plates and incubated for 24 h, followed by treatment with 100 *μ*m H_2_O_2_ or NAC for 2 h. The cells were then rinsed with XF base medium 3 times and cultured in a 37°C incubator without CO_2_ for an additional 1 h prior to the bioenergetics measurement. The oligomycin, carbonyl cyanide 4-(trifluoromethoxy)phenylhydrazone (FCCP), and rotenone/antimycin A were added subsequently. The oxygen consumption rate (OCR) of iPSCs was measured following the process of the XF96 Extracellular flux analyzer (Agilent Technologies Inc.) [23]. The default Mix-Wait-Measure times were 3 min–0 min–3 min. The basal respiration, ATP production, maximal respiration, and proton leak were measured. The experiment was performed with five replicates.

### 2.14. Effect of ROS on iPSC-Mediated Apoptosis Inhibition and Induced Proliferation of Renal Epithelial Cells That Were Not in Direct Contact

HK2 apoptosis was examined using annexin V/PI staining. Briefly, the human tubule epithelial cells (HK2) were plated in 12-well plates (50,000 cells/well) and cultured for 24 h followed by hypoxic conditions (1% O_2_, 5% CO_2_, 37°C) for 2 h. Transwells with a 0.4 *μ*m pore size (Corning Inc., ME), a size permeable for cell-released substances but not for intact cells, were placed on top of the culture wells. iPSCs or iPSCs pretreated with 100 *μ*m H_2_O_2_ or NAC for 2 h were gently added into the upper chamber of Transwell (1 × 10^5^ cells/well) and cultured under normoxic conditions for 24 h and 48 h. After the Transwell inserts along with iPSCs in them were removed, HK2 cells were incubated with annexin V-fluorescein isothiocyanate and propidium iodide for 15 min. The apoptosis rate of the HK2 cells was measured by a flow cytometer (BD FACSCelesta™, BD Biosciences, CA).

Cell viability of HK2 cells was determined using the Cell Counting Kit-8 (CCK-8) (Dojindo Laboratories, Kumamoto, Japan) according to the manufacturer's instructions. The cells were treated the same as that described above for apoptosis measurement in 96-well plates (3000 HK2 cells/well). One thousand cells/well of iPSCs were placed in the upper chamber of the insert. Cell viability curves of HK2 cells were constructed from absorbance measurements at 450 nm using a microplate spectrophotometer (Synergy H1, BioTek Instruments Inc., Winooski, VT).

### 2.15. Statistical Analysis

Unpaired two-tailed Student's *t*-test was used to compare the differences between two groups. Data are represented as mean ± standard error of the mean.

## 3. Results

### 3.1. ROS Are Necessary for iPSCs to Improve Kidney Morphology

**Figure 1:**
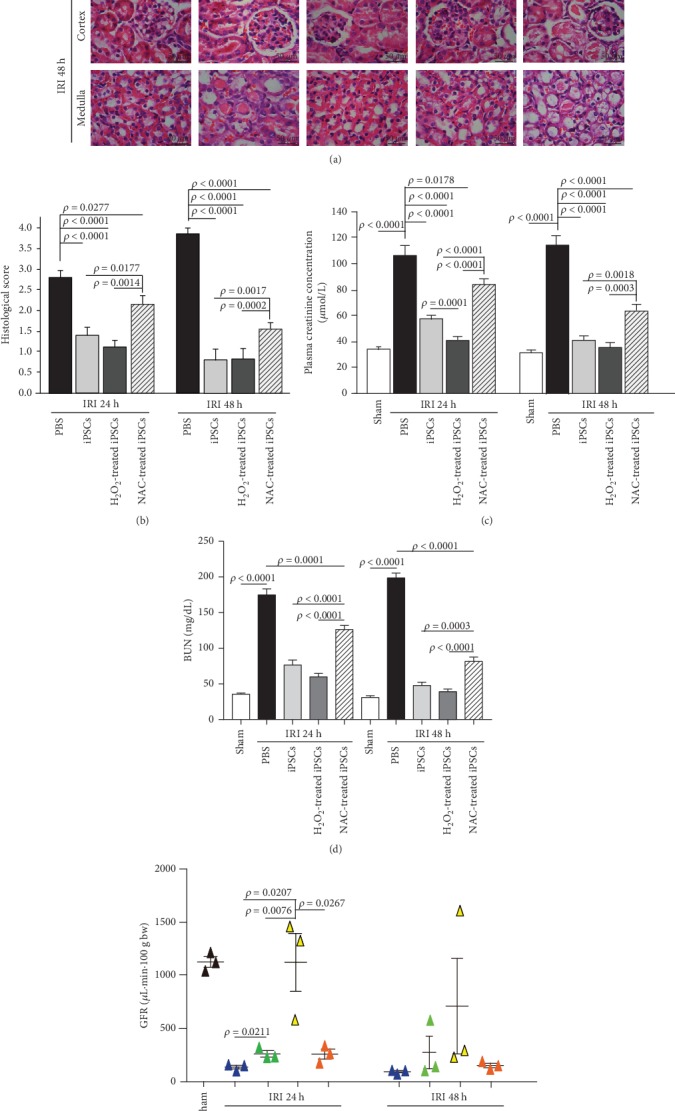
ROS mediated iPSC improvement of renal morphology and function. (a) C57BL/6 male mice were subjected to 50 min of kidney ischemia followed by 24 h and 48 h reperfusion, respectively. PBS, iPSCs, or iPSCs pretreated with H_2_O_2_ or NAC were injected via the tail vein. Kidney tissues were fixed, embedded, sectioned, and stained with H&E. Histological changes included tubular cell necrosis, cytoplasmic vacuole formation, hemorrhage, and tubular dilatation were assessed with a maximum score of 4. (b) Semiquantitative analysis of morphological changes. (c) Serum creatinine (*μ*mol/L) and (d) blood urea nitrogen (BUN; mg/dL) were measured at 24 h and 48 h after reperfusion; *n* = 10. (e) Glomerular filtration rate measured by transcutaneous decay of FITC-sinistrin; *n* = 3.

The extent of kidney IRI was assessed by a semiquantitative histological scoring system of 0–4 with normal tissue at 0. iPSCs decreased renal morphological disorder scores in mice receiving PBS from 2.80 ± 0.17 at 24 h and 3.85 ± 0.13 at 48 h to 1.40 ± 0.19 and 0.85 ± 0.11, respectively. Pretreating iPSCs with N-acetyl-l-cysteine (NAC, a panoramic ROS inhibitor) decreased the score to a level between those of nonpretreated iPSCs and without iPSC treatment. No differences were observed between iPSCs without pretreatment and iPSCs briefly pretreated with H_2_O_2_ (100 *μ*mol/L, 2 h) before being injected via the tail vein (Figures 1(a) and 1(b)).

### 3.2. ROS Are Necessary for iPSCs to Improve Kidney Function

The renal function of mice was assessed based on blood creatinine and BUN levels. Kidney IRI increased serum creatinine levels to 105.9 ± 7.49 *μ*mol/L at 24 h and 113.99 ± 6.87 *μ*mol/L at 48 h from a sham control level of 33.68 ± 2.23 *μ*mol/L (Figure 1(c)). A bolus intravenous injection of iPSCs or H_2_O_2_ treated-iPSCs (3 × 10^6^ cells/kg body weight in 100 *μ*L) decreased serum creatinine levels by 46.3% and 62.6% at 24 h, respectively, and to normal levels at 48 h after reperfusion. NAC-pretreated iPSCs decreased creatinine levels by 20.9% at 24 h and 44.7% at 48 h, respectively (Figure 1(c)).

Kidney IRI increased the BUN levels to 174.99 ± 8.29 mg/dL at 24 h and 198.692 ± 6.57 mg/dL at 48 h from the sham control levels of 35.79 ± 1.48 mg/dL and 31.03 ± 2.51 mg/dL at 24 and 48 h, respectively (Figure 1(d)). A bolus intravenous injection of iPSCs and H_2_O_2_-treated iPSCs (3 × 10^6^ cells/kg body weight in 100 *μ*L) decreased BUN by 56.2% and 65.8% at 24 h after reperfusion, respectively, and to normal levels at 48 h after reperfusion. NAC-pretreated iPSCs decreased BUN by 27.9% and 58.9% at 24 and 48 h, respectively (Figure 1(d)).

Kidney IRI decreased the GFR (*μ*L/min/100 g body weight) from 1128.84 ± 50.34 (sham control) to 138.85 ± 17.76 and 94.36 ± 10.85 at 24 and 48 h, respectively (Figure 1(e)). A tail bolus intravenous injection of iPSCs and NAC-treated iPSCs (3 × 10^6^ cells/kg body weight in 100 *μ*L) increased GFR by 89.40% and 88.19% at 24 h after reperfusion, respectively. In addition, H_2_O_2_-pretreated iPSCs resulted in 7.10-fold and 6.61-fold increase at 24 h and 48 h after kidney IRI, respectively (Figure 1(e)).

### 3.3. iPSCs Trafficked to the Injured Kidney Tissue in an ROS-Dependent Manner

**Figure 2:**
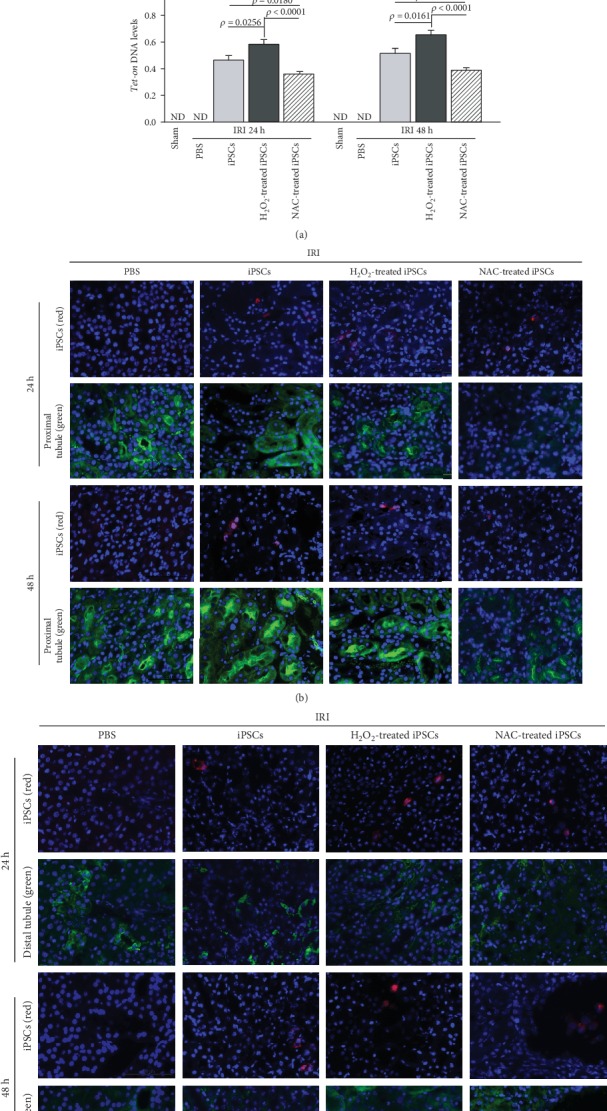
ROS promoted while NAC inhibited iPSC trafficking to the injured kidney. (a) Quantitative PCR analysis of *Tet-on* and *GAPDH* genes in the injured kidney. *Tet-on* was detected in the kidneys of mice receiving iPSCs with or without H_2_O_2_ or NAC pretreatment but not from mice receiving *PBS* or undergoing sham operations. Relative amount of *Tet-on* DNA over *GAPDH* DNA in the injured kidney; *n* = 10. (b, c) Injected PKH26-labeled iPSCs were not positive for makers of proximal (b) or distal (c) tubular cells. Red: injected iPSCs. Green: proximal (b) or distal (c) tubular cells; *n* = 5.

The number of cells homed to the injured kidney tissue was measured by quantitative real-time PCR of the *Tet-on* gene. The *Tet-on* gene was carried to iPSCs by a lentivirus vector during iPSC induction to control the expression of transfected genes. It presented in the injected cells but not in recipient mice. The PCR results confirmed the presence of the administered cells in the injured kidney tissue. The pretreatment of iPSCs with H_2_O_2_ increased *Tet-on* gene levels significantly. In contrast, NAC pretreatment decreased *Tet-on* gene levels by 39.9% and 40.6% at 24 and 48 h after reperfusion, respectively (Figure 2(a)).

### 3.4. Intravenously Delivered iPSCs Trafficked to IRI Kidney but Failed to Differentiate into Renal Epithelial Cells

The iPSCs were observed in the injured kidney at 24 and 48 h after being injected via tail vein. However, iPSCs trafficked in IRI kidney failed to differentiate into aquaporin-positive proximal (Figure 2(b)) or calbindin D28K-positive distal renal tubular epithelial cells (Figure 2(c)).

### 3.5. Transplanted iPSCs Reduced the Expression of Inflammatory Cytokines in IRI Kidneys

**Figure 3:**
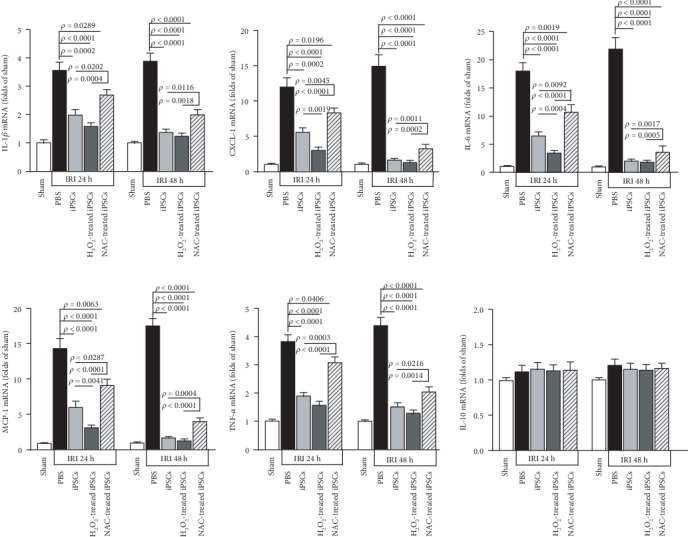
ROS pretreatment promoted while NAC inhibited iPSC capability to decrease inflammatory cytokines in injured kidney. The mRNA levels of interleukin-1*β*, CXCL1, interleukin-6, MCP-1, TNF-*α*, and interleukin-10 were measured by quantitative reverse transcription PCR at 24 h and 48 h after renal IRI; *n* = 10.

Inflammatory cytokine levels are indicators of active lesions. Measured by RT-qPCR, IRI increased the expression levels of interleukin-1*β*, CXCL1, interleukin-6, MCP-1, and TNF-*α* mRNA levels in the kidneys by 2.56-, 11.01-, 16.89-, 13.39-, and 2.77-fold from the levels present in sham controls at 24 h after IRI (Figure 3). The levels increased further at 48 h after IRI. IL-10 did not change significantly. At 24 h after IRI, the levels of these increased cytokines decreased significantly upon intravenous administration of iPSCs. H_2_O_2_ pretreatment promoted this inhibitory effect of iPSCs. At 48 h after IRI and cell treatment, all cytokine levels in IRI kidneys in groups other than the NAC-pretreated iPSCs were similar to those in kidneys of sham controls. The NAC-pretreated iPSCs were significantly less effective in decreasing cytokine expression in IRI kidneys than untreated iPSCs (Figure 3).

### 3.6. NAC Decreased While H_2_O_2_ Increased ROS in iPSCs

**Figure 4:**
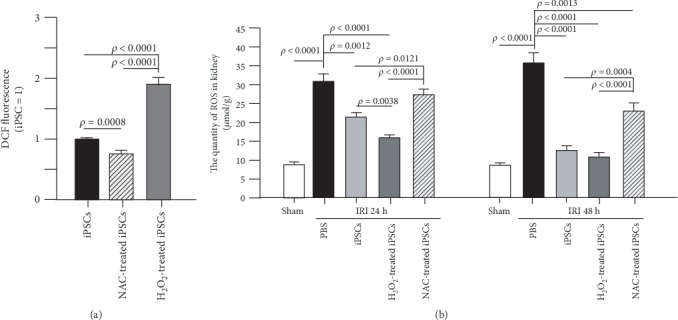
ROS pretreatment promoted while NAC pretreatment inhibited iPSC capability to decrease ROS levels in the injured kidney. (a) ROS pretreatment increased while NAC pretreatment decreased ROS levels inside iPSCs; *n* = 5. (b) ROS pretreatment promoted while NAC pretreatment decreased the capability of iPSCs to decrease IRI-increased ROS levels in kidney; *n* = 10.

When iPSCs were pretreated with H_2_O_2_ (100 *μ*M) or NAC (100 *μ*M) for 30 min, H_2_O_2_ increased while NAC decreased intracellular ROS when compared with the untreated iPSCs (Figure 4(a)).

### 3.7. H_2_O_2_-Pretreated iPSCs Decreased ROS in IRI Kidney Tissue More Significantly than Unpretreated or NAC-Pretreated iPSCs

Using DHE as a fluorescent probe for the detection of ROS generation, ROS activity in kidney tissue was measured. At 24 h after IRI, unpretreated iPSCs decreased IRI-increased ROS from 30.56 ± 1.92 to 21.14 ± 1.52 (*μ*mol/g tissue) in kidney tissue. The inhibitory effect of H_2_O_2_-pretreated iPSCs was more significant than that of unpretreated iPSCs. NAC-pretreated iPSCs had no significant effect. At 48 h after IRI, both unpretreated and H_2_O_2_-pretreated iPSCs decreased kidney ROS activity to a level similar to that of sham controls (8.80 ± 0.61 *μ*mol/g), while ROS in untreated IRI kidney remained as high as 35.40 ± 2.63 *μ*mol/g. NAC-pretreated iPSCs had a less significant effect in decreasing ROS activity in IRI kidney (22.74 ± 2.05 *μ*mol/g) (Figure 4(b)).

### 3.8. ROS Regulated iPSC Viability

**Figure 5:**
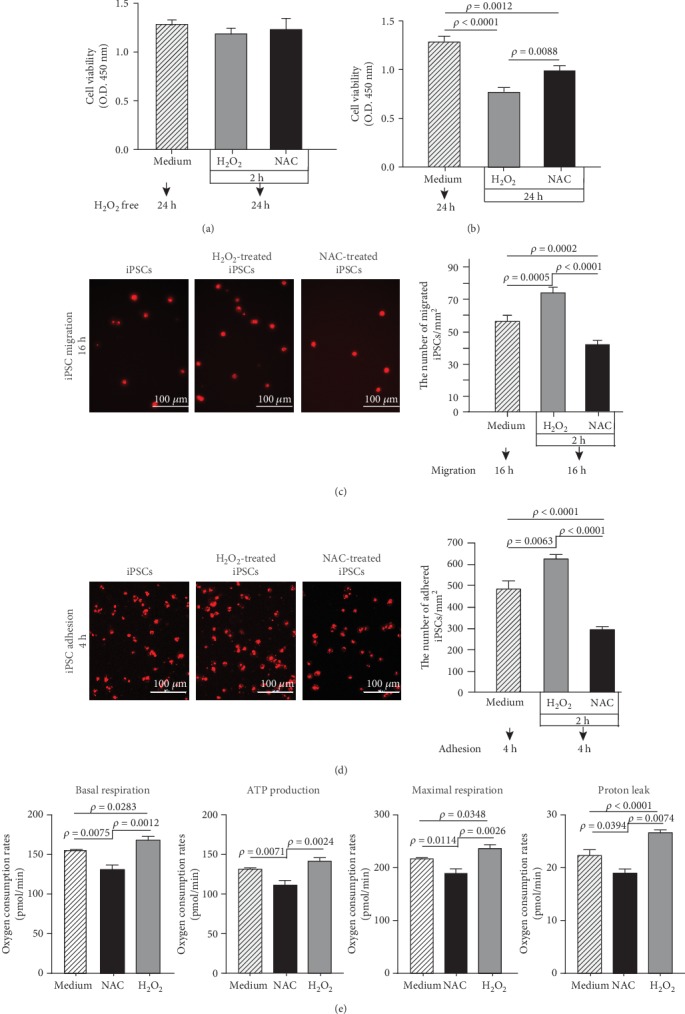
ROS regulated iPSC viability, adhesion, migration, and mitochondrial bioenergetics. (a) Temporary treatment (2 h) of iPSCs with ROS or NAC did not affect iPSC viability (*n* = 5). (b) Persistent treatment (24 h) of iPSCs with ROS and NAC decreased iPSC viability (*n* = 5). (c) ROS pretreatment promoted while NAC pretreatment inhibited iPSC migration through porous membranes (*n* = 10). (d) ROS pretreatment promoted while NAC pretreatment inhibited iPSC adhesion to extracellular matrix-coated culture surfaces (*n* = 10). (e) Basal oxygen consumption rate, ATP production, maximal respiration rate, and proton leak respiration of iPSCs, H_2_O_2_-treated iPSCs, or NAC-treated iPSCs (*n* = 5).

Temporary pretreatment of iPSCs with 100 *μ*M H_2_O_2_ or NAC for 2 h did not change cell viability. In contrast, persistent treatment with 100 *μ*M H_2_O_2_ or NAC for 24 h decreased iPSC viability (Figures 5(a) and 5(b)).

### 3.9. Temporary H_2_O_2_ Treatment Increased iPSC Adherence to Culture Surfaces Coated with Extracellular Matrix Molecules and Enhanced Migration across Permeable Membranes

Temporary pretreatment of iPSCs with 100 *μ*M H_2_O_2_ for 2 h enhanced their migration across Transwell™ membranes (Figure 5(c)) and increased iPSC adherence to culture surfaces coated with extracellular matrix while the effect of NAC was inhibitory (Figure 5(d)).

### 3.10. H_2_O_2_ Treatment Promoted Mitochondrial Bioenergetics in iPSCs

H_2_O_2_ treatment of iPSCs resulted in a nearly 7.6-19.4% increase in mitochondrial metabolic activity of the cells, represented by basal respiration, maximal respiration, proton leak, and ATP production, respectively. In contrast, NAC treatment resulted in 12.5-15% decrease in the mitochondrial metabolic parameters (Figure 5(e)).

### 3.11. iPSCs Inhibited Apoptosis and Promoted Proliferation of Renal Epithelial Cells without Direct Contact in a ROS-Dependent Manner

**Figure 6:**
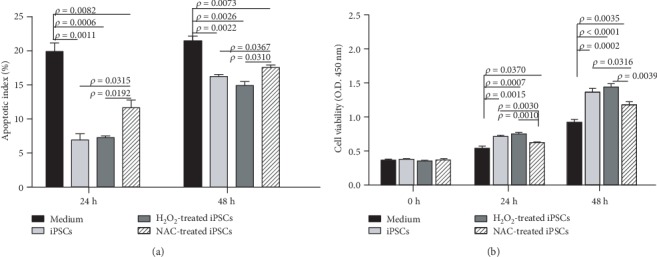
iPSCs inhibited apoptosis and promoted proliferation of renal epithelial cells without direct contact in an ROS-dependent manner. (a) ROS pretreatment promoted capability of iPSC-released substances to decrease apoptosis of HK2 cells subjected to hypoxia/normoxia. (b) ROS pretreatment increased the capability of iPSC-released substances to promote HK2 cell proliferation; *n* = 5.

Renal proximal epithelial cells (HK2) were subjected to hypoxia for 2 h followed by normoxia for 24 or 48 h. During normoxia, iPSCs were allowed to interact indirectly with the HK2 cells across the membrane of Transwell insert. Addition of iPSCs, H_2_O_2_-pretreated iPSCs or NAC-pretreated iPSCs into the upper chamber of the Transwell insert decreased HK2 apoptosis rate at 24 h from 19.90 ± 1.26% in the control (only culture medium added) to 6.95 ± 0.92, 7.25 ± 0.25, and 11.65 ± 1.13%, respectively. At 48 h, the apoptosis rates were 21.68 ± 0.72% in control, 16.24 ± 0.29 with iPSCs, 15.42 ± 0.59 with H_2_O_2_-pretreated iPSCs, and 17.64 ± 0.35% with NAC-pretreated iPSCs (Figure 6(a)).

Substances released from iPSCs and H_2_O_2_-pretreated iPSCs promoted HK2 proliferation by 32.28% and 39.25% at 24 h and by 47.66% and 55.93% at 48 h over those without indirect interaction of iPSCs, respectively. The substances released from NAC-pretreated iPSCs promoted proliferation by 15.67% and 27.47% at 24 and 48 h, respectively (Figure 6(b)).

## 4. Discussion

The need for new therapeutic strategies for AKI is critical due to its high morbidity, mortality, and currently limited therapeutic options [24, 25]. Furthermore, AKI is a harbinger of chronic kidney disease and end-stage renal disease [26]; hence, its treatment offers both short-term and long-term benefits. Stem cell-based therapies for kidney diseases have attracted considerable attention [27]. However, the engraftment of adult stem cells in injured tubules and their development into functional renal tissues are insufficient to repair acute renal injury [28, 29]. Moreover, the use of embryonic stem cells is restricted by ethical issues as well as allogenic mismatch. The patient's own somatic cells can be transformed into iPSCs, thereby rendering the potential for patient and disease specificity. iPSCs may be an ideal source for cell-based therapies.

In the present study, we demonstrated that iPSCs administered intravenously 2 h following kidney IRI-improved kidney function and morphology and decreased kidney inflammation. PCR assessment of the *Tet-on* gene in iPSCs, which is not present in recipient mice, showed that the injected cells were recruited to injured kidneys. The trafficking of injected iPSCs was associated with improved kidney histology and function as well as decreased inflammatory cytokines. These results demonstrated that intravenous administration of iPSCs decreased IRI in kidneys.

Although the iPSCs decreased kidney IRI, they did not differentiate into renal proximal or distal epithelial cells. Thus, endo/paracrine may be the major mechanism by which iPSCs ameliorated kidney IRI. This is consistent with our in vitro result that iPSCs promoted proliferation and inhibited apoptosis of renal epithelial cells.

The role of ROS in cells can be described as a double-edged sword. On the one hand, they are key side products during the production of ATP in mitochondria and many important hormones such epinephrine and noradrenaline, as well as in intracellular detoxification. On the other hand, overproduction or leaking of ROS from precisely controlled intracellular compartments results in cell injury. For example, ROS production is one of the major mechanisms of IRI [30], while ROS inhibitors reduce kidney IRI [31, 32]. ROS, along with other inflammatory mediators, activate leukocytes and endothelial cells to facilitate leukocyte infiltration in inflammatory lesions [33]. However, whether ROS inside iPSCs regulate the therapeutic effect of iPSCs *in vivo* is unknown.

We previously found that 24 h persistent treatment of iPSCs with high concentrations of H_2_O_2_ injured iPSCs [34]. In this study, we found that 2 h temporary treatment with H_2_O_2_ did not affect iPSC viability. Instead, H_2_O_2_ pretreatment increased while NAC pretreatment decreased the OCR and OCR for ATP production of iPSC mitochondria. Mitochondrial metabolism provides energy for most cell functions. Meanwhile, H_2_O_2_ pretreatment increased while NAC pretreatment decreased iPSC adherence and migration—processes necessary for cell homing. Although iPSCs encounter an IRI-induced oxidative microenvironment after iPSC extravasation and inhabitation in injured kidney, the IRI-increased intrakidney ROS concentration is significantly lower than the concentration harmful to iPSCs. In fact, iPSC treatment decreased ROS in IRI kidneys. Pretreatment of iPSCs with H_2_O_2_ before iPSCs were injected intravenously to treat kidney IRI significantly increased iPSC trafficking to the injured kidney, rendering an increased therapeutic effect when compared with untreated iPSCs. In contrast, the effect of NAC pretreatment was inhibition.

## 5. Conclusion

The systemic administration of iPSCs is a promising therapeutic strategy for acute kidney diseases, and it may be through endo/paracrine effects instead of by differentiating into renal tubular cells. Intracellular ROS is necessary for normal mitochondrial metabolism of iPSCs and for the cells to engraft and treat IRI-injured kidney. Moderate increases in intracellular ROS promote iPSC engraftment and the ensuing therapeutic function. Moreover, extensive antioxidants should not be administered simultaneously with these cells (Supplemental Fig. [Supplementary-material supplementary-material-1]).

## Data Availability

The data generated or analyzed of this study are included in the linked data website (http://homepage.fudan.edu.cn/xiangmeng/oxidative-medicine-and-cellular-longevity/).

## Supplementary Materials

Supplementary Materials Supplemental Figure 1: kidney blood flow. Representative images of kidney blood flow before, during, and after (10 min) kidney ischemia/reperfusion injury. Supplemental Figure 2: graphical abstract. Intracellular ROS are necessary for iPSCs to engraft and treat IRI-injured kidney. Moderate increases in intracellular ROS promote iPSC engraftment and the ensuing therapeutic function. Supplemental Table 1: detailed information on primer sequences and key resources.
