# Supplementary material for: Intracellular Reactive Oxygen Species Mediate the Therapeutic Effect of Induced Pluripotent Stem Cells for Acute Kidney Injury
Source: Oxid Med Cell Longev. 2020 Mar 26;2020:1609638. doi: 10.1155/2020/1609638 (PMC7136790; doi:10.1155/2020/1609638)
Supplement: Supplementary Materials — Supplemental Figure 1: kidney blood flow. Representative images of kidney blood flow before, during, and after (10 min) kidney ischemia/reperfusion injury. Supplemental Figure 2: graphical abstract. Intracellular ROS are necessary for iPSCs to engraft and treat IRI-injured kidney. Moderate increases in intracellular ROS promote iPSC engraftment and the ensuing therapeutic function. Supplemental Table 1: detailed information on primer sequences and key resources. [file 1609638.f1.zip › mat.1609638.v2.pdf]

Supplemental figure legends

Supplemental figure 1. Kidney blood flow. Representative images of kidney blood flow before, during and after (10 min) kidney ischemia/reperfusion injury.

Supplemental figure 2. Graphical abstract. Intracellular ROS are necessary for iPSCs to engraft and treat IRI injured kidney. Moderate increases in intracellular ROS promote iPSC engraftment and the ensuing therapeutic function.

Supplemental Figure 1

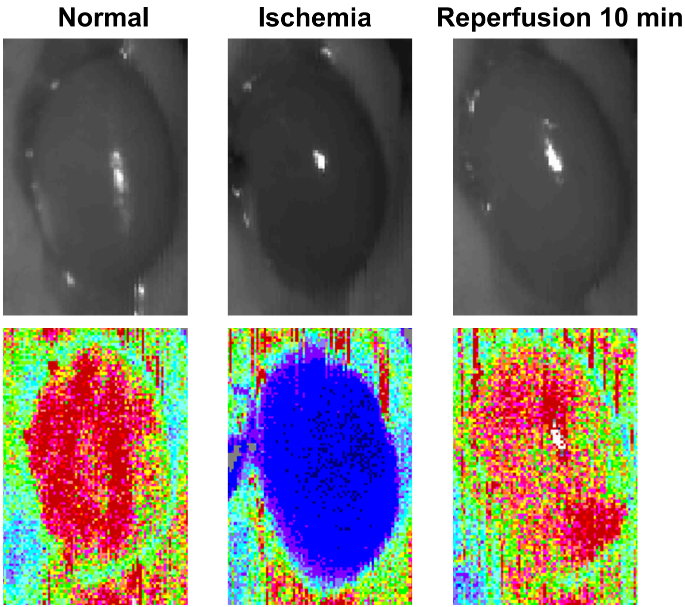

Kidney blood flow. Representative images of kidney blood flow before, during and after (10 min) kidney ischemia/reperfusion injury.

Supplemental Figure 2

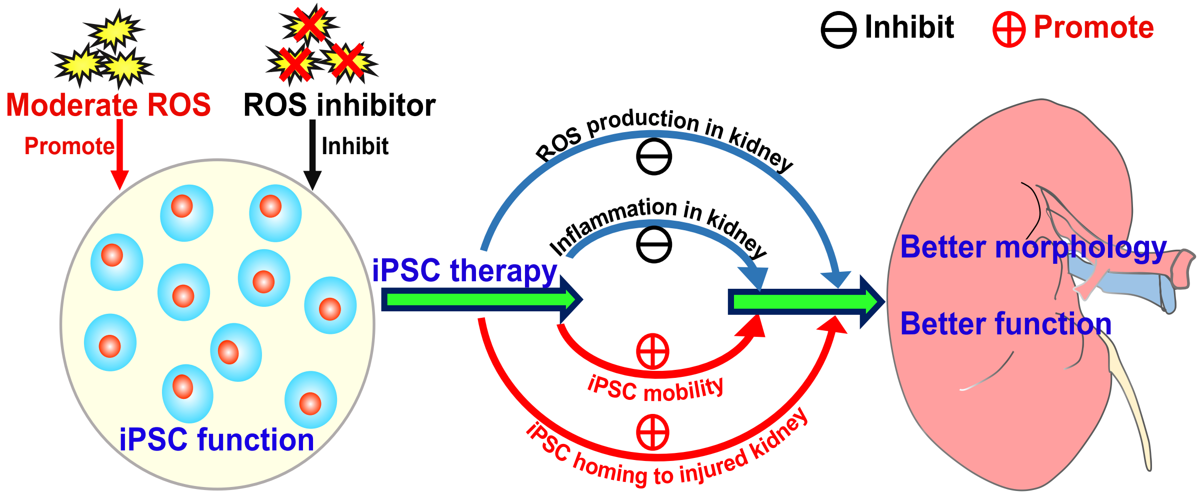

Graphical abstract. Intracellular ROS are necessary for iPSCs to engraft and treat IRI injured kidney. Moderate increases in intracellular ROS promote iPSC engraftment and the ensuing therapeutic function.

Supplemental Table 1 . Detailed information on primer sequences and key resources

Primers		Primer pair sequences	Amplicon size
IL-1 β (mouse)	forward	TGCCACCTTTTGACAGTGATG	220 bp
	reverse	AAGGTCCACGGGAAAGACAC	
CXCL-1 (mouse)	forward	ACCGAAGTCATAGCCACACTC	113 bp
	reverse	CTCCGTTACTTGGGGACACC	
IL-6 (mouse)	forward	GCCTTCTTGGGACTGATGCT	181 bp
	reverse	TGCCATTGCACAACTCTTTTCT	
MCP-1 (mouse)	forward	AGCTGGAGCTGAGGAGATTA	100 bp
	reverse	GTCCTCAGAACCTCTGTCCG	
TNF-α (mouse)	forward	CCCTCACACTCACAAACCAC	133 bp
	reverse	ACAAGGTACAACCCATCGGC	
IL-10 (mouse)	forward	GGTTGCCAAGCCTTATCGGA	159 bp
	reverse	GGGGAGAAATCGATGACAGC	
GAPDH (mouse)	forward	GTATTGGGCGCCTGGTC	201bp
	reverse	GCTCCTGGAAGATGGTGATG	
Tet-on	forward	AGCACAACTACGCCGCACCC	402bp
	reverse	ATGCACCAGAGTTTCGAAGC	
18s (mouse)	forward	GAGAAACGGCTACCACATCC	170 bp
	reverse	CACCAGACTTGCCCTCCA	

Chemicals	Source	Identifier
Creatinine assay kit	Jiancheng Bioengineering Institute, Nanjing, China	Cat#C011-1-1
BUN assay kit	Jiancheng Bioengineering Institute, Nanjing, China	Cat#C013-2-1
Non-traumatic microvessel clamp	S&T AG, Neuhausen, Switzerland	Size B-1 V
Optimum cutting temperature compound (OCT)	Sakura Finetek Inc., Tokyo, Japan	Cat#4583
Dihydroethidium	Beyotime Biotechnology Inc., Shanghai, China	Cat#S0063

Cell counting kit-8	Dojindo Molecular Technologies Inc., Shanghai, China	Cat#LC617
Mounting medium, anti-fading	Solarbio Science & Technology Ltd, Beijing, China	Cat#S2100
Hydrogen peroxide solution	Sigma-Aldrich Co., Saint Louis, MO, USA	Cat#323381
Trypsin-ethylene diamine tetraacetic acid	Sigma-Aldrich Co., Saint Louis, MO, USA	Cat#T4049
N-acetylcysteine	Sigma-Aldrich Co., Saint Louis, MO, USA	Cat#BP907
PKH26 red fluorescent cell linker kit	Sigma-Aldrich Co., Saint Louis, MO, USA	Cat#MIDI26
Fetal bovine serum	Thermo Fisher Scientific Inc., MA, USA	Cat#10100147
Matrigel	BD Biosciences Inc., San Jose, USA	Cat#354277
Transwell	Corning Inc., NY, USA	Cat#3464，Cat#3401
Sucrose	Sinopharm Chemical Reagent Ltd, Shanghai, China	Cat#10021418
SYBR Green PCR Master Mix	Thermo Fisher Scientific Inc., MA, USA	Cat#4309155
Paraformaldehyde	Sigma-Aldrich Co., Saint Louis, MO, USA	Cat#158127
Gelatin	Sigma-Aldrich Co., Saint Louis, MO, USA	Cat#V900863
Dulbecco's modified Eagle's medium	Thermo Fisher Scientific Inc., MA, USA	Cat#10565018
L-Glutamine	Thermo Fisher Scientific Inc., MA, USA	Cat#25030081
Sodium pyruvate	Thermo Fisher Scientific Inc., MA, USA	Cat#11360070
Non-essential amino acids	Thermo Fisher Scientific Inc., MA, USA	Cat#11140050
β-Mercaptoethanol	Sigma-Aldrich Co., Saint Louis, MO, USA	Cat#97622-10ML
Leukemia inhibitory factor	Merck Millipore Ltd., Darmstadt, Germany.	Cat#LIF2005
Penicillin-streptomycin	Thermo Fisher Scientific Inc., MA, USA	Cat#15140163

Pentobarbital sodium	Sigma-Aldrich Co., Saint Louis, MO, USA	Cat#P3761
DreamTaq Green PCR Master Mix	Thermo Fisher Scientific Inc., MA, USA	Cat#K1082
TRIzol Reagent	Thermo Fisher Scientific Inc., MA, USA	Cat#15596026
DNAzol^{TM} Reagent	Thermo Fisher Scientific Inc., MA, USA	Cat#10503027
Monoclonal anti-aquaporin 1	Abcam Inc., Cambridge, UK	ab-9566
Monoclonal calbindin D28K	SantaCruz Biotechnology Inc., CA, USA	sc-365360
Agilent Seahorse XF cell Mitochondrial stress test kit	Agilent Technologies Inc., Santa Clara, CA,US	103015-100
**Animals**	**Organisms/Strains**	
Mouse: C57BL/6	Shanghai Laboratory Animal Ltd, Shanghai, China	
**Instruments**	**Source**	**Model**
Freezing microtome	Leica Biosystems, Wetzlar, Germany	CM1950
Microplate reader	BioTek Instruments Inc., Winooski, VT, USA	Synergy H1
CFX96^{TM} Real-Time system	Bio-Rad Laboratories Inc., Berkeley, CA, USA	CFX96^{TM} Optics Module
Tanon 3500 gel imaging system	Tanon Science & Technology Ltd, Shanghai, China	Tanon 3500
Tissuelyzer	JingXin Industrial Development Ltd, Shanghai, China	JXFSTPRP-24
Fluorescence microscope	Leica Microsystems, Wetzlar, Germany	DMi8
MoorLDI2-2 laser doppler imaging system	Moor Instruments Ltd., Devon, UK	MoorLDI2-HIR
Transdermal GFR	NIC-Kidney, Mannheim Pharma & Diagnostics	SN:UD1211

monitor	GmbgH, Mannheim, Germany	
F96 extracellular flux analyzer	Agilent Technologies Inc, CA, USA	Seahorse XFe/XF
